# Coupling and Elastic Loading Affect the Active Response by the Inner Ear Hair Cell Bundles

**DOI:** 10.1371/journal.pone.0033862

**Published:** 2012-03-27

**Authors:** Clark Elliott Strimbu, Lea Fredrickson-Hemsing, Dolores Bozovic

**Affiliations:** 1 Department of Physics & Astronomy, University of California Los Angeles, Los Angeles, California, United States of America; 2 California NanoSystems Institute, University of California Los Angeles, Los Angeles, California, United States of America; University of Salamanca- Institute for Neuroscience of Castille and Leon and Medical School, Spain

## Abstract

Active hair bundle motility has been proposed to underlie the amplification mechanism in the auditory endorgans of non-mammals and in the vestibular systems of all vertebrates, and to constitute a crucial component of cochlear amplification in mammals. We used semi-intact *in vitro* preparations of the bullfrog sacculus to study the effects of elastic mechanical loading on both natively coupled and freely oscillating hair bundles. For the latter, we attached glass fibers of different stiffness to the stereocilia and observed the induced changes in the spontaneous bundle movement. When driven with sinusoidal deflections, hair bundles displayed phase-locked response indicative of an Arnold Tongue, with the frequency selectivity highest at low amplitudes and decreasing under stronger stimulation. A striking broadening of the mode-locked response was seen with increasing stiffness of the load, until approximate impedance matching, where the phase-locked response remained flat over the physiological range of frequencies. When the otolithic membrane was left intact atop the preparation, the natural loading of the bundles likewise decreased their frequency selectivity with respect to that observed in freely oscillating bundles. To probe for signatures of the active process under natural loading and coupling conditions, we applied transient mechanical stimuli to the otolithic membrane. Following the pulses, the underlying bundles displayed active movement in the opposite direction, analogous to the twitches observed in individual cells. Tracking features in the otolithic membrane indicated that it moved in phase with the bundles. Hence, synchronous active motility evoked in the system of coupled hair bundles by external input is sufficient to displace large overlying structures.

## Introduction

At thresholds of auditory detection, mechanical sensitivity can reach into the sub-nanometer regime [1]. The inner ear contains hair cells that detect these minute displacements induced by air-borne or ground-borne vibrations and transduce them into electrical signals [2], [3]. To achieve this detection, hair cells utilize an active amplification process to enhance oscillations evoked by low-level stimulation and sharpen the frequency selectivity of the response. The active process in hair cell response depends on multiple biological mechanisms or combinations of mechanisms, variant among species [4]–[6]. The mechanical response of the auditory endorgans has been captured by systems of nonlinear dynamic equations [7]–[10], which include a compressive nonlinearity, observed experimentally both in the movements evoked in stereociliary bundles *in vitro* [11] and in the vibrations measured *in vivo* in the basilar membrane of the mammalian cochlea [12]. Equations of hair bundle mechanics were shown to support a Hopf bifurcation; dependent on the value of a dynamic parameter, the bundle may be quiescent or may exhibit spontaneous oscillation [13].

**Figure 1:**
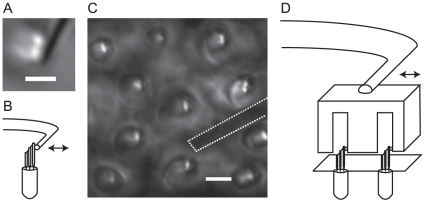
Experimental setup. (A) Top-down view of a freestanding hair bundle with a glass probe (black line positioned vertically across the image) attached to the tallest row of stereocillia. Because of light piping, the stereocillia appear much brighter than the background. Scale bar: 2.5 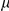
m. (B) Schematic diagram showing a side view of a hair bundle with a probe attached. The piezoelectric actuator displaces the probe's base in the direction of the bundle's axis of sensitivity indicated by the arrow in the figure. (C) Top-down view of hair bundles coupled to the otolithic membrane. The pits in the membrane into which the bundles protrude give rise to the bright ellipses around each bundle. The shadow of the probe's tip has been highlighted with the dashed line. Scale bar: 5 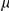
m. (D) Schematic diagram of a side view of hair bundles coupled to the otolithic membrane and stimulated with a probe. In the actual experiment, the probe's tip is embedded a few microns into the otolithic membrane. Note than in C and D, the probes have not been drawn to scale; in particular, the cantilever arms are typically a few hundred microns in length.

Mechanically decoupled hair bundles from the bullfrog sacculus exhibit spontaneous oscillations *in vitro* [14]–[17]. *In vivo*, most of the 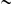
2500 hair cell bundles of the sacculus are coupled to the otolithic membrane, a 25–30 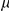
m thick extracellular matrix that is anchored to the apical surface of the epithelium by an 8–10 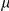
m thick layer of columnar filaments [18], [19]. Variation in the thickness of the otolithic membrane creates fluid-filled “pits” above the stereociliary bundles (Fig. 1 C and D). Simultaneous tracking of bundles and adjacent edges of these pits indicated that the filamentous connections attaching the otolithic membrane to the tips of the stereocilia are significantly stiffer than the bundles [20]. The dominant compliance in the load is therefore the patch of overlying membrane, shown to be roughly impedance-matched to the bundles [21]. Hair bundles of the frog sacculus do not oscillate spontaneously *in vitro* when the otolithic membrane is left attached to the epithelium, suggesting that the mechanical loading tunes the hair bundles into the quiescent rather than the oscillatory regime [22]. To explore the effects of loading on individual hair cell bundles, we attached glass rods of varying stiffness to the stereocilia.

In addition to loading the hair bundles, the overlying membrane also couples them. Experiments performed *in vivo* on the cochlea [12] have shown that it supports frequency-selective amplification. Recent evidence has extended these observations to the semicircular canals, showing that hair bundles coupled to the cupula exhibit active amplification *in vivo* [23]. Numerical simulations have suggested that coupling even a modest number of identical oscillators in a 2-dimensional array could significantly enhance the sensitivity and sharpen the frequency tuning of the system [24]. *In vitro*, decoupled hair bundles of the bullfrog sacculus display a broad but peaked response around their characteristic frequencies, with 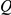-factors 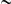
1 [11]. To investigate the effects of loading and coupling on the response characteristics of the sacculus as a whole, we measured the movements evoked in hair bundles attached to the overlying otolithic membrane and explored the frequency dependence of the response.

Apart from spontaneous oscillation, the active process in decoupled hair bundles manifests itself in rapid active movements or “twitches” in response to transient step stimuli [15], [25], [26]. Similar active movements were seen in the bundles of outer hair cells from the rat cochlea when the cells were rapidly depolarized [27]. In saccular and turtle papillar cells, the twitches were shown to be correlated with the transduction current, sensitive to the extracellular calcium concentration, affected by the resting position of the bundle, and abolished when the transduction channels are blocked [15], [25], [26]. This active movement has been proposed as a potential amplification mechanism for non-mammalian hair bundles. We explored whether the twitch could still be observed in hair bundles of the coupled system. As the otolithic membrane was shown to synchronize the response of hair bundles across the epithelium [28], we also studied whether the active twitch evokes a phase-locked movement in the overlying membrane.

## Results

### Effects of elastic loading

#### Spontaneous oscillations in decoupled hair bundles

**Figure 2:**
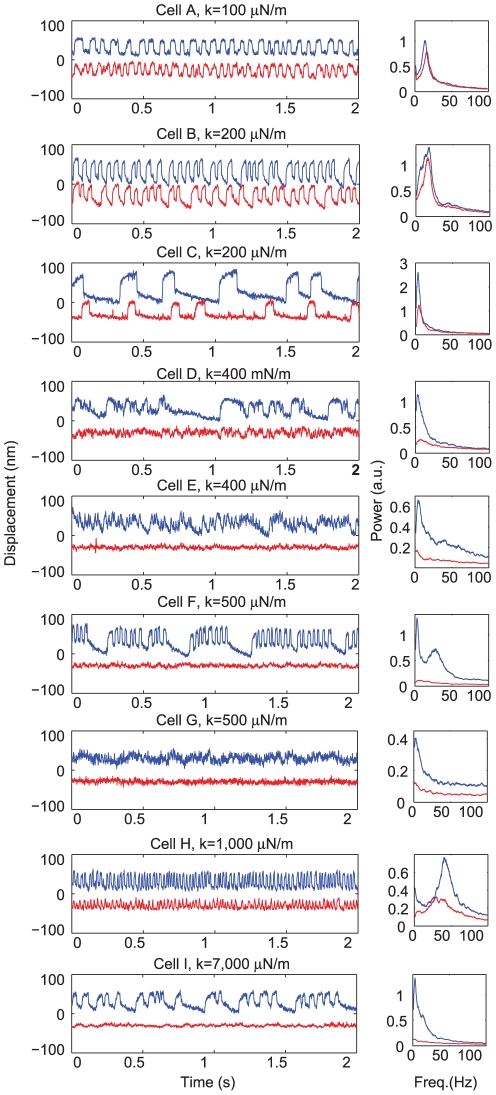
Change in the profile of spontaneous oscillation upon mechanical loading. Time-dependent traces of spontaneous oscillations of hair bundles obtained from 9 cells before (blue) and after (red) attachment of a glass probe. Traces are arranged from the least stiff load (100 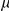
N/m), with cell A shown in the top panel, to the most stiff load (7,000 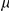
N/m), with cell I shown in the bottom panel, and intermediate stiffnesses shown in between. For clarity, the traces have been plotted with an arbitrary offset. Small panels to the right of the traces show the power spectra (in arbitrary units) of bundle motion before and after probe attachment. The spectra display almost complete flattening with imposition of heavier loads. Traces were recorded at 500 fps.

The complementary metal oxide semiconductor (CMOS) imaging and motion-tracking software described in the Methods section allowed us to record spontaneous oscillations exhibited by hair cell bundles in the absence of an external load. Spontaneous oscillations characteristically occur in the 10–80 Hz frequency range, peaked around 30 Hz, with amplitudes of 20–100 nm. Figure 2 shows typical examples of innate motility observed in stereociliary bundles before (blue) and after (red) the attachment of glass fibers of various stiffnesses. Imposing an external load qualitatively changed the oscillation profiles. Loading with relatively elastic probes reduced the amplitude and increased the characteristic frequency; stiffer loads completely arrested the oscillation. These changes in the oscillation profile were consistently observed in all 70 cells measured. Suppression of oscillation typically occurred under loads of 500–1000 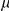
N/m, comparable to natural loading [21]. In control experiments, gentamicin, a pharmacological agent known to block transduction channels, removed innate oscillation of both loaded and unloaded bundles (data not shown).

#### Frequency selectivity of the evoked response

To fully characterize the effects of loading on the response function of a hair bundle, we measured the motility evoked by mechanical stimuli, applied over the physiological range of the sacculus. Glass fibers were attached to the tallest row of stereocilia and used to deliver lateral sinusoidal displacements at increasing amplitudes and in discrete frequency increments. All of the hair bundles recorded exhibited spontaneous oscillations prior to the attachment of the stimulus probes.

**Figure 3:**
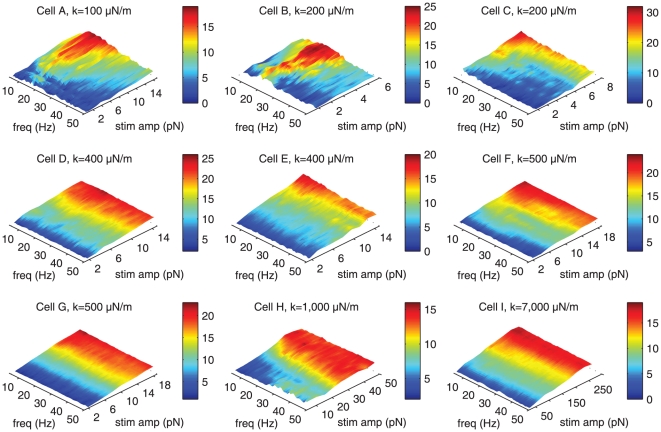
Frequency selectivity of the phase-locked response under mechanical loading. A discrete frequency sweep was applied to each bundle with the attached glass probe. Each sweep ranged from 5–50 Hz in 1 Hz increments, with 5 cycles presented at each frequency. The frequency trains were applied at increasing amplitudes. The phase-locked response was extracted for each frequency and amplitude and displayed in a color-coded plot, with the range shown to the right of each panel. Under light loading (100–200 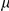
N/m), the bundles exhibited tuning around their characteristic frequencies. Under stiffer loading (400 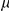
N/m and higher), the bundles displayed no tuning. Note that the plots contain only 1∶1 mode-locking; higher-order modes were not included. Traces were recorded at 500 fps.

Upon loading with fibers that were elastic with respect to the hair bundles (100–200 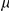
N/m), low-amplitude stimulation evoked a frequency-selective response, while higher-amplitude stimuli led to entrainment over the full range of frequencies (Figure 3A and B). The triangular shape apparent in the 3-dimensional plots is indicative of an Arnold Tongue, the phase-locking predicted for numerous instances of nonlinear dynamical systems [29], [30]. Frequency tuning was seen to broaden with increasing stiffness of the imposed load (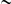
400 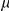
N/m, Figure 3C and D). At approximate impedance matching (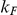 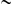
0.5–1 mN/m) corresponding to natural conditions and higher levels of loading, the frequency tuning was completely abolished (Figure 3E–I).

### Effects of coupling of hair bundles to the otolithic membrane

#### Broadening of the frequency tuning

Spontaneous oscillations observed in decoupled hair bundles were shown to be mutually uncorrelated and to display a broad distribution of characteristic frequencies, with a peak 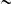
30 Hz [31]. When entrained by an external mechanical stimuli, the bundles display frequency selectivity with 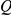-factors on the order of 1 [11]. To test the effects of natural loading and coupling on the frequency selectivity of the saccular epithelium as a whole, we used semi-intact preparations in which the otolithic membrane was left attached to the stereociliary bundles. Glass fibers were brought into contact with the membrane and used to deliver lateral sinusoidal displacements. Deflections evoked in the underlying hair bundles were measured as described in the Methods section. A total of 48 bundles were recorded from 7 preparations.

**Figure 4:**
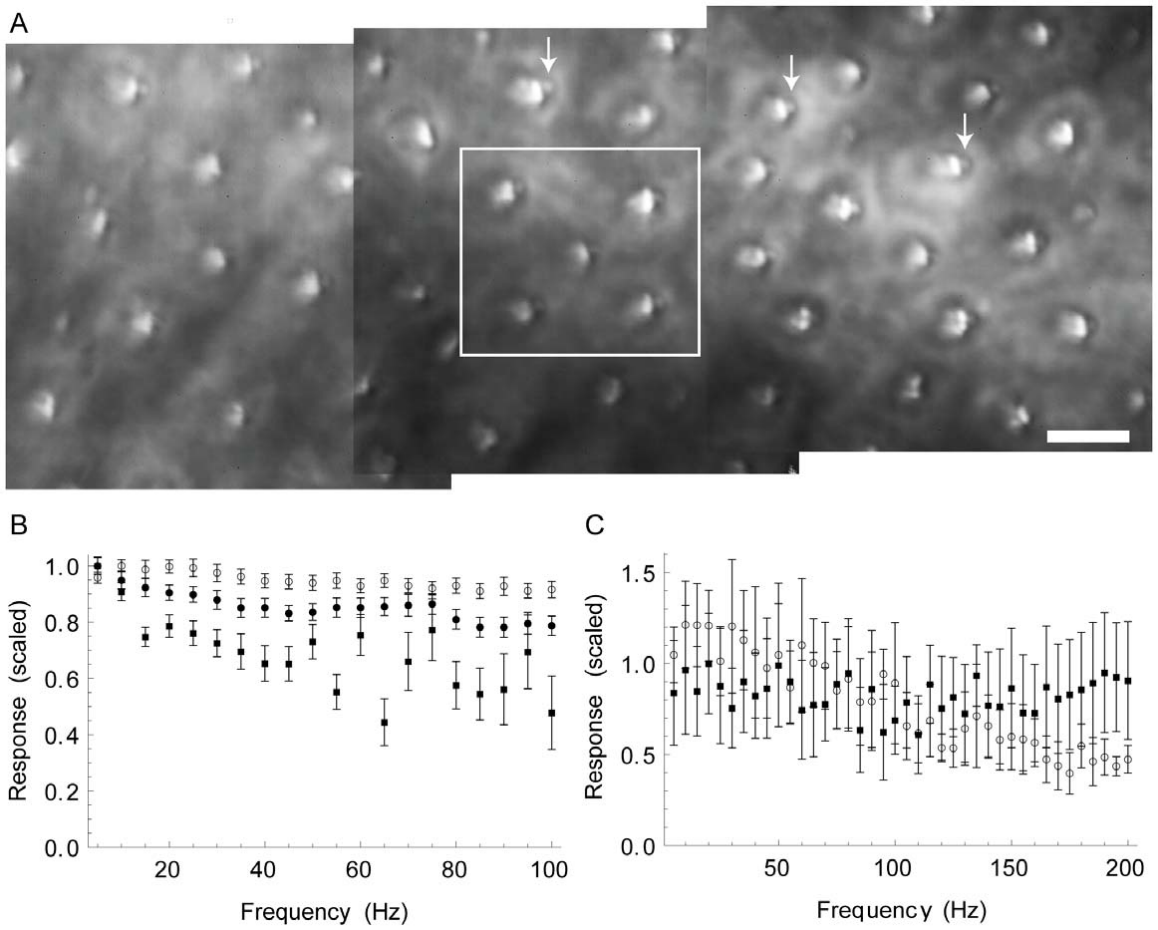
Coupling of active hair bundles by the otolithic membrane broadens the response to sinusoidal stimulation. (A) A top-down view of a 150
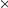
50 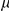
m area of the saccular epithelium with the otolithic membrane removed. The linear response function averaged over the four bundles in the corners of the white square is shown in panel C (the central bundle was partially occluded by an otolith). The tallest row of stereocilia appear as elongated bright features. The kinociliary bulbs (indicated by arrows) appear as grey circles to the right of the stereocilia. Scale bar: 10 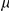
m. (B) Normalized linear response functions for three hair bundles coupled to the otolithic membrane. The mean and standard deviation at each frequency were computed from a digitally resampled trace on a cycle-by-cycle basis over the 1 s recording. When the otolithic membrane was removed from this sample, 84% of bundles exhibited spontaneous oscillations demonstrating that the active process was maintained throughout the measurement. C. Ensemble-averaged (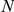 = 4) linear response function of bundles coupled to the otolithic membrane from the preparation shown in panel A. Filled squares: the average response was essentially flat across the physiological range of frequencies. Open circles: Blocking the transduction channels with 20 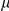
M gentamicin had little effect on the response at low frequencies; the response was modestly reduced at frequencies above 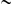
150 Hz. With the otolithic membrane removed, 90% of the bundles in this preparation oscillated spontaneously. All recordings were taken at 1000 fps.

Figure 4B shows the normalized phase-locked amplitude of three selected hair bundles coupled by the otolithic membrane and stimulated in parallel. Although considerable variation was observed in the peak response within and across preparations, the frequency tuning was consistently broad over the entire physiological range of frequencies. The majority of bundles showed very modest tuning around the lowest frequencies sent (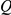-factor 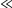
1). Only 2 bundles had higher 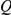-factors (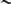
2). Six bundles exhibited notches at higher frequencies, the occurrence of which was however not apparent in the ensemble-averaged sensitivity. No tuning was observed in the response averaged over multiple coupled hair bundles (filled circles in Fig. 4C).

On four preparations, the measurements were repeated with the transduction channels blocked by gentamicin. Little effect was observed in either the sensitivity or frequency selectivity of the response. For the preparation shown in Figure 4C, a modest falloff of the response function was observed at frequencies above 150 Hz when the transduction channels were blocked with gentamicin; in other preparations, there was little or no change across the full frequency range in the presence of the channel blocker. As a control, the otolithic membrane was removed after the measurements, and spontaneous oscillations were assessed over a large area (up to 150
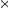
50 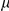
m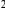) of the exposed epithelium. On five of the seven preparations measured, the majority of the bundles oscillated spontaneously, confirming that the active process was maintained throughout the experiment. The two remaining preparations showed fewer spontaneously oscillating bundles; comparably broad tuning was observed in all of the preparations.

#### Active Hair Bundle Movement

**Figure 5:**
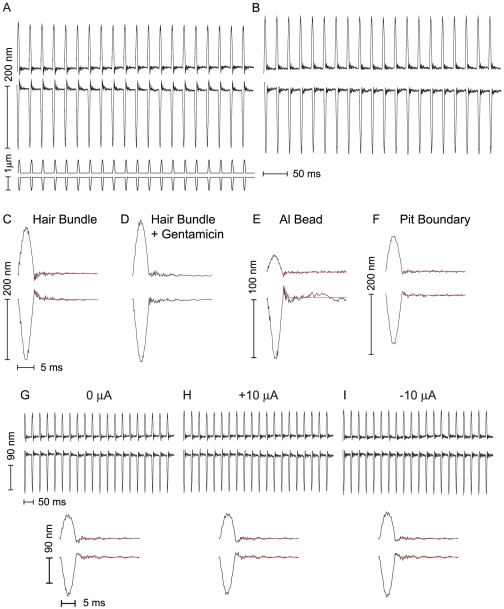
Active hair bundle movement under the otolithic membrane. (A)Traces of twitches in underlying hair bundles evoked by a series of 5 ms pulses delivered to the otolithic membrane in the excitatory (top) and inhibitory (bottom) directions. The command signals were half-cycles of a 100 Hz sine wave and are shown below the traces. (B) With the transduction channels blocked with 20 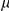
M gentamicin, the active movement was abolished. The probe had a stiffness of 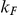 = 1200 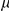
N/m. (C) and (D) Average traces for the bundle movement shown in panels A and B. (E) The averaged traces obtained from 1 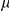
m aluminum oxide beads dispersed on the otolithic membrane of the same preparation. (F) Average traces of a pit boundary measured in a different preparation. In both E and F, the otolithic membrane was entrained by the active bundle movement at the end of the applied pulse. The stimulus fiber used on the sample in panel F had a stiffness of 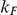 = 1400 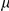
N/m. In C, E, and F the exponential fits (red lines) from which the recovery times were extracted, have been overlaid on the traces. (G) Response of a hair bundle which showed a 15 nm twitch in response to sinusoidal pulses. (H) +10 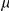
A transepithelial electric current had little effect on either phase-locked amplitude or the twitch for both stimuli. (I) −10 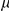
A current reduced the magnitude of the twitch to 10 nm for positive deflections but had a negligible effect for negative displacements. In G, H, and I the average traces have been plotted below the raw data.

**Figure 6:**
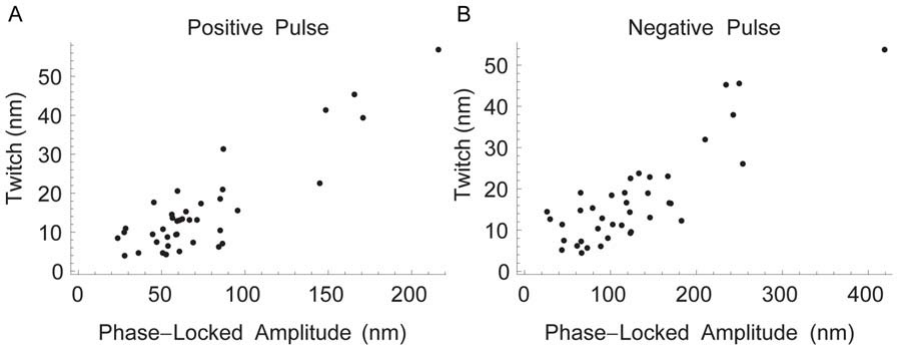
The magnitude of the twitch scales linearly with phase-locked amplitude. (A) and (B) Scatter plots of the active movement or twitch versus the phase-locked amplitude for positive and negative deflections. Data have been pooled from five preparations. All recordings were taken at 5000 fps.

Mechanically decoupled hair bundles of the frog sacculus [15], [26] and the turtle papilla [25] have been shown to exhibit rapid active movements, termed “twitches”, in response to transient mechanical stimuli. To test whether active movements can be evoked from stereociliary bundles coupled to the otolithic membrane, we used compliant (with respect to the stiffness of a hair bundle and its neighboring extracellular filaments [21]) probes to deliver pulses to the overlying membrane and measured the response of the underlying hair bundles. When the membrane was stimulated by sinusoidal pulses in either the excitatory or the inhibitory direction, the attached hair bundles displayed a complex active response: after following the stimulus during deflection, the bundles showed considerable motion in the opposite direction immediately subsequent to the pulse, returning to their equilibrium position with an approximately exponential relaxation. Figure 5A shows the response of a bundle to pulses in both directions. The observed temporal profile of the active movement was similar to the twitch measured in individual free-standing hair bundles. The mean time constant for recovery from deflections was 0.6
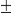
0.2 ms for the excitatory and 0.9
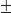
0.4 ms for the inhibitory direction (Fig. 5 C), with a statistically significant difference (Student's *t*-Test, 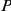 = 0.0007, 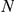 = 30). The magnitude of the active movement was proportional to the phase-locked amplitude (Fig. 6) for pulses in both the excitatory and inhibitory direction.

A number of control experiments were performed to confirm that the observed motion was dependent on the active process. First, no hydrodynamic artifact was observed in the motion of the probes' tips over the stiffness range used in these experiments: when the probes were pulsed in water, their tips perfectly followed the command signal sent to the base. Second, in some preparations, only a fraction of hair bundles exhibited the enhanced biphasic response, further confirming that the twitches were not a ubiquitous hydrodynamic effect. Third, the twitch-like response was abolished by the introduction of gentamicin into the endolymph solution bathing the apical surface (Fig. 5 B and D).

Our results indicate that synchronized active twitches evoked by the applied pulses generate sufficient force to induce motion in the overlying otolithic membrane. When the preparations provided sufficient optical contrast between the pit boundaries and the surrounding patch of membrane, we tracked movement of the membrane adjacent to each hair bundle. On other preparations, we dispersed 1 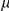
m aluminum beads onto the surface of the membrane and tracked their motion. Traces for both the beads (Fig. 5E) and the pit boundaries (Fig. 5F) showed that the active twitch followed the applied pulse, demonstrating that the bundles can generate sufficient force to move the otolithic membrane and that the movement is not limited to hair bundles or to a localized deformation near the pit boundaries. As with the bundle movement (Fig. 5B), twitches in the response of the otolithic membrane were likewise abolished when the transduction channels were blocked with gentamicin (data not shown).

To test whether modulation of the transduction process, including influx of calcium, affects the active response we applied steady-state currents across the epithelium [32]. We recorded active movements from 8 hair bundles on two preparations under both positive and negative current offsets. Figure 5G–I shows the response of a hair bundle to sinusoidal pulses under these conditions. With the applied current, a number of bundles showed some modulation in the phase-locked response; we therefore characterized the effect of the transepithelial current by measuring the change in the ratio of the twitch to phase-locked amplitude under different current offsets. For pulses in the excitatory direction, +10 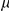
A current had a weak effect on the twitch to phase-locked amplitude ratio. The twitch to amplitude ratio was more strongly reduced by −10 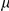
A current with the ratio dropping from 0.19 to 0.13. For pulses in the inhibitory direction, positive and negative currents had a negligible effect on the twitch to amplitude ratio.

## Discussion

In a prior study performed on preparations in which the otolithic membrane was left attached to the hair cell bundles, we observed that spontaneous oscillation was suppressed by the presence of the overlying extracellular matrix [22]. Applying a localized mechanical stimulus evoked a phase-locked response in hair bundles across most of the saccular epithelium, implying that the membrane imposes strong coupling. The entrainment between the bundles and the adjacent pits in the membrane revealed no measurable phase lags, indicating that the coupling between the two is elastic rather than viscous. We estimated a lower bound for the stiffness of the filamentous links connecting the stereociliary bundle to the otolithic membrane to be at least several-fold higher than the bundle or the corresponding patch of membrane, demonstrating that the connections do not introduce a compliant element, and hence an additional degree of freedom, into the system [20].

To evaluate the effects of loading on an individual bundle, we attached probes of various stiffness. We particularly explored the regime where compliance of the probes was comparable to that of the otolithic membrane, approximating the natural conditions. Even under light loading, spontaneous oscillations exhibited a qualitatively different profile, indicative of a change in the internal dynamic state of the system. Stronger loading, at approximately impedance-matched and higher stiffness, consistently suppressed innate oscillation and flattened the frequency selectivity of the bundle.

Theoretical studies have proposed that hair cells contain an internal control parameter by which they can self-tune to the vicinity of the critical point separating the oscillatory from the quiescent regime [33]. Mechanical loading can therefore provide a means by which the active process of the bundle could be rendered quiescent, with any variation allowing the bundle to self-tune towards or away from the oscillatory regime. External elements, the otolithic membrane and the connecting filaments, are unlikely to change their stiffness *in vivo* and thus do not provide likely candidates for the biological control parameter. However, any modulation of the stiffness of the stereociliary bundle itself would change the effective relative loading, and hence constitute a potential control mechanism. A number of cellular processes have been proposed that could affect the compliance of the bundle: myosin motor activity [26], [34], calcium-dependent relaxation [32], variable stiffness of the gating spring [35], or a voltage-sensitive element [36].

Apart from loading, the otolithic membrane also provides coupling between the bundles. Additionally, individual hair bundles of the sacculus display a lack of tonotopy and a broad distribution of frequencies of spontaneous oscillation [31]. Theoretical studies have shown that strong coupling between nonlinear oscillators of different characteristic frequencies can lead to suppression of oscillation [37]. Consistent with this prediction, our previous work demonstrated suppression of spontaneous oscillations in the coupled system [22]. We observed a decrease in the frequency tuning when the hair bundles are coupled to the otolithic membrane in contrast to predictions based on numerical simulations [24], [38]. The response was flattened across the physiological range. Electrophysiological recordings performed on the VIIIth cranial nerve of different frog species *in vivo* indicate a high sensitivity to small stimuli (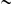
1 Å) but poor frequency selectivity (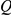-factors below 1) [39], [40]. The broad tuning is also consistent with innervation patterns in the sacculus, where up to hundreds of hair cells can be innervated by a single nerve fiber [41].

When the bundles are phase-locked by an applied signal, the power generated by the hair cells' active process was shown to be sufficient to evoke movement in the otolithic membrane. Hence, while spontaneous synchronization of innate oscillation seems to be precluded in the sacculus by the broad distribution of characteristic frequencies and the elastic loading, collective action by hair bundles can still be triggered by an external pulse. We note that *in vivo*, the hair cells of the sacculus are likely stimulated *en bloc* by the motion of the otoconial mass. Strong coupling between hair cells may therefore enable the sacculus to detect minute transient stimuli.

## Materials and Methods

### Biological Preparation

Prior to performing experiments, all animal-handling protocols were approved by the UCLA Chancellor's Animal Research Committee (Protocol Number ARC 2006-043-13C) in accordance with federal and state guidelines. Adult bullfrogs (*Rana catesbeina*) were anesthetized with 200 to 600 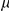
L of 50 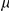
g/mL sodium pentobarbital and euthanized. The inner ears were excised and saccular macculae were separated from the accessory structures and mounted in a two-compartment chamber. For experiments on free-standing hair bundles, dissections were performed in artificial perilymph (110 mM 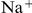, 2 mM 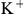, 1.5 mM 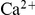, 118 mM 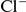, 3 mM d-glucose, 1 mM sodium pyruvate, 1 mM creatine, and 5 mM HEPES). For preparations on which the otolithic membrane was left intact, dissections were performed in a modified artificial endolymph (2 mM Na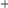, 117.5 mM K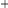, 0.25 mM Ca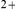, 118 mM Cl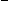, 3 mM d-glucose, 5 mM HEPES, 110 mM n-methyl-d-glucamine (NMDG)). The use of this solution avoided exposing the membrane to artificially high concentrations of extracellular calcium during the dissection, which may alter the stiffness of the membrane [42]. Once mounted in the experimental chamber, the basolateral surface of the preparation was immersed in artificial perilymph and the apical surface was bathed in artificial endolymph (117.5 mM 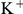, 2 mM 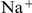, 0.25 mM 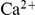, 118 mM 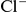, 3 mM d-glucose, and 5 mM HEPES). All solutions were titrated to pH 7.3, had their osmolarities adjusted with sucrose to 230 mOs/L, and were freshly oxygenated prior to use. To record freely oscillating hair bundles, the otolithic membrane was removed with a human eyelash following a 7–8 minute digestion at room temperature in 15 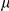
g/mL collagenase Type 1A–S (Sigma-Aldrich) in 4 mM Ca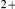 endolymph. All dissections and experiments were performed at room temperature.

For control experiments, 10–20 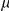
M gentamicin sulfate (Sigma-Aldrich) was added to artificial endolymph and the fluid in the apical compartment was replaced with a fluid exchange; multiple exchanges were performed to ensure complete replacement. For experiments with the otolithic membrane left intact, measurements were taken after a 5 minute incubation period to allow the gentamicin to diffuse through the membrane; for experiments on free-standing hair bundles, spontaneous oscillations were arrested following the introduction of gentamicin. Motion of the otolithic membrane was measured either by following the edges of the pits, or by tracking beads on top of the membrane. For the latter, 1 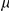
m aluminum oxide beads (Microspheres-Nanospheres, Cold Springs, NY) were diluted to a concentration of 0.05% and dispersed into the endolymph. Following a 5 minute incubation, the apical surface was rinsed with endolymph to remove beads that did not adhere to the membrane.

### Imaging

Epithelia were imaged with an upright microscope (Olympus B51X) using a 20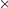 water-immersion objective (XLUMPLFL20XW, .95 N.A.) and illuminated by a X-Cite 120 W metal halogenide lamp. Images were further magnified to 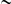
400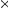 with a double-Gauss, variable-focus lens and projected onto a CMOS camera (Photron SA 1.1). The microscope and camera were mounted on a vibration-isolation table (Technical Manufacturing Corp.) and placed inside a sound-isolation booth (Industrial Acoustic Co.). Most experiments were performed on hair cells between the striola and the abneural edge of the epithelium. Samples were rotated in the microscope stage so as to align the hair-bundles' axes of sensitivity along the horizontal direction. High-speed video recordings were taken at 12-bit pixel depth and recorded at 500, 1000, or 5,000 frames-per-second (fps). The spatial scale was calibrated with a 600 linepair per mm Ronchi ruling (Edmund Optics) to be 53 nm/pixel. Motion traces were extracted from the video records as described previously [31]. At the frame rates used in this study, raw traces had a root-mean-square (rms) noise level of 2–3 nm, with the variation due to different optical contrast in different areas of the epithelium. For experiments in which averaging was performed over multiple presentations of a periodic stimulus, the noise level was reduced to 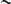
1 nm.

### Mechanical Stimulation

Borosilicate glass capillaries were pulled with a micropipette puller (Sutter Instruments Co. Flaming/Brown P-97). The tapers were cut with surgical scissors, then pulled again at 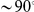 with a microforge. The cantilever arms were a few hundred 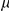
m in length and 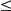
1 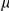
m in diameter. Probes were sputter-coated with 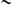
100 nm of gold-palladium (Anatech Hummer 6.2) to enhance optical contrast. The stiffness and viscous drag coefficient of each fiber were measured by recording the brownian motion of its tip in water and fitting the power spectrum to a Lorenzian distribution. For single-cell measurements, probes' stiffnesses (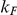) ranged from 100 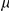
N/m to 7 mN/m. For experiments in which the cells remained coupled to the otolithic membrane, probe stiffnesses ranged from 0.8–1.5 mN/m. The viscous drag coefficients 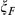 were on the order of 100 nNs/m. The fibers' corner frequencies 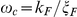 were on the order of 1 kHz, well above the frequency range of the bullfrog sacculus.

For measurements of individual hair bundles, the probe tips were dipped in 2 mg/mL concanavilin A (Sigma-Aldrich) to improve adhesion to the stereocilia. Probes were mounted on a piezoelectric stack actuator (PiezoJenna PA 4/12), the position of which was controlled with an electric micromanipulator. Probes were positioned above the preparation and brought into contact with either the tallest row of stereocillia or the otolithic membrane. The piezoelectric stimulator was controlled by a function generator (Tektronix AFG 3022), which simultaneously triggered the CMOS camera recording. Figures 1A and B show, respectively, a top-down microscopic image and a schematic diagram of the probe attached to the bundle. Figures 1C and D describe the stimulus setup used for experiments done on preparations with intact otolithic membranes.

For experiments on tuning in the coupled system, stimulus frequencies were varied across the physiological range (up to 200 Hz) in 5 or 10 Hz increments, at fixed stimulus amplitudes, either 250 or 500 nm. The frequency selectivity of the evoked motion in the underlying hair bundles was characterized by the dimensionless response function 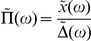, where 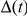 is the command signal sent to the base of the probe, 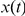 is the induced displacement of the bundle, and 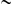 denotes the Fourier transform. For each preparation, the response function was calculated in two ways. We first computed the time-averaged response functions 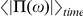 for each hair bundle by digitally resampling the trace and computing the response on a cycle-by-cycle basis. Secondly, we computed the ensemble average response function 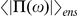 by averaging over all bundles recorded in parallel over the entire length of the recording. At 400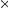 magnification, we were able to record up to 5 hair bundles in parallel. On two preparations, we increased the number of bundles recorded simultaneously by lowering the magnification to 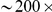. The response functions of individual hair bundles and the ensemble average on these 2 samples showed the same stereotypic broad shape. Following each measurement, we removed the otolithic membrane and recorded a 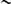
150
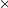
50 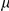
m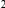 region of the epithelium, typically covering 30–40 cells. For most of the preparations, the majority of hair bundles (up to 90%) exhibited spontaneous oscillations, indicating that the active process was maintained throughout the measurement.

### Active Movement

Pulse trains (5 ms half-sinusoidal pulses spaced 20–95 ms apart) were delivered to the overlying otolithic membrane, and hair bundle motion was recorded at 1000–5000 fps. To improve the signal-to-noise ratio for the transient response, each trace was averaged over presentations of the pulse train. At both frame rates, we were able to measure the magnitude of the active response in the averaged trace. The phase-locked amplitude of each bundle was measured by fitting the first 5 ms of the trace to a sine wave. For the bundles recorded at 5000 fps, we measured the relaxation time following the twitch from the remainder of the trace. For 30/39 bundles recorded the recovery was well described by a single exponential decay (Fig. 5) fit to the final 20 ms of the trace. Samples in which a minority of hair bundles showed the active movement or “twitch” were excluded from further analysis.

A linear stimulus isolator (World Precision Instruments A395) was used to pass constant electric currents though the saccular epithelium. Freshly chlorided silver wires were placed in both the apical and basolateral compartments of the chamber, and the current values were controlled with a function generator. To avoid a large transient at the onset and end of each step stimulus, the command signals were low-pass filtered at 10 Hz. Positive currents are defined as passing from the apical to basal sides of the epithelium. Mechanical pulse trains were delivered the the otolithic membrane as described above, with the transepithelial current clamped at 0, +10, and −10 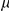
A. Several seconds were allowed to elapse between the onset of the current and the mechanical stimulus.
